# Developmental milestones and cognitive trajectories in school-aged children with 16p11.2 deletion

**DOI:** 10.1186/s11689-025-09615-7

**Published:** 2025-06-19

**Authors:** Jente Verbesselt, Jeroen Breckpot, Inge Zink, Ann Swillen

**Affiliations:** 1 Department of Human Genetics, Catholic University Leuven, Leuven, Belgium; 2 Research Group Experimental Oto-Rhino-Laryngology (ExpORL), Department of Neurosciences, Leuven Brain Institute, Catholic University Leuven, Leuven, Belgium; 3 Children’s Hospital of Philadelphia, Philadelphia, USA; 4 Centre for Human Genetics, University Hospitals Leuven, Leuven, Belgium; 5 Department of Oto-Rhino-Laryngology, Head & Neck Surgery, MUCLA, University Hospitals Leuven, Leuven, Belgium

**Keywords:** 16p11.2 deletion syndrome, Copy number variants, Early development, Developmental trajectories, Deep phenotyping, Cognition

## Abstract

**Background:**

16p11.2 deletion syndrome (16p11.2DS) is a recurrent CNV that occurs de novo in approximately 70% of cases and confers risk for neurodevelopmental disorders, including intellectual disability (ID) and autism spectrum disorders (ASD). The current study focusses on developmental milestones, cognitive profiles and longitudinal cognitive trajectories.

**Methods:**

In-person assessments, digital medical records and parental interviews on developmental history of 24 children (5–16 years) with a confirmed BP4-BP5 16p11.2DS were reviewed and analysed for developmental milestones (motor, language, continence). Standardised intelligence tests were administered in all children, and longitudinal IQ-data were available for a subgroup (79%, 19/24).

**Results:**

Motor, language, and continence milestones were delayed. Average IQ was in the borderline range (IQ 71) with 46% (11/24) having borderline IQ (IQ 70–84). Both intra- and interindividual variability were found across the five cognitive domains with significant discrepancies between verbal and non-verbal skills in 55% (11/20). Longitudinal IQ-data indicate that school-aged children with 16p11.2DS perform statistically significantly lower at the second time point (*p* < 0.001) with 58% showing a growing into deficit trajectory.

**Conclusion:**

Delayed motor, language and continence milestones are common in 16p11.2DS carriers. School-aged children with 16p11.2DS show increasing cognitive impairments over time, pointing to the need for early diagnosis, regular cognitive follow-up and individualised intervention. The high prevalence of disharmonic IQ-profiles highlights the importance of expanding the focus beyond full-scale IQ (FSIQ) outcomes. Future studies in larger cohorts including carrier relatives are needed to gain more insight into the penetrance and phenotypic variability of 16p11.2DS.

**Supplementary Information:**

The online version contains supplementary material available at 10.1186/s11689-025-09615-7.

## Introduction

The proximal 16p11.2 deletion syndrome (16p11.2DS) defined by breakpoints 4 and 5, and encompassing 29 genes, is one of the most frequent copy number variants (CNVs) in the general population [10, 17, 46]. The deletion occurs de novo in approximately 70% of cases [29]. While existing literature outlines a spectrum of clinical manifestations, including reduced penetrance and variable expressivity, several gaps persist in our understanding of the neurodevelopmental and cognitive aspects and trajectories of 16p11.2DS.

Previous research has focused on clinical features associated with the deletion, such as overweight/obesity and seizures, and neurodevelopmental features, including psychiatric issues, speech-language and motor impairments, and autism spectrum disorders (ASD) [5, 6, 8, 11, 13, 17, 20, 25, 29, 35, 37, 54]. Despite consistent reports of developmental delays in 16p11.2DS, exact data on motor and language milestones and time of bladder control have not been characterised before. In addition, the question raises whether the time point of reaching developmental milestones could be informative of later cognitive functioning.

Cognitive capacities exhibit a broad range from average IQ to intellectual disability (ID) with average full-scale IQ (FSIQ) falling within the borderline range (IQ 70–84) [3, 13, 19, 20, 25–27]. Verbal and non-verbal IQ (VIQ – NVIQ) scores are within the same range, with on average, slightly higher NVIQ scores [3, 8, 13, 16, 27, 28]. In one study, VIQ was significantly lower than NVIQ [54]. Moreover, a trend towards lower FSIQ in patients with inherited 16p11.2DS (FSIQ 74) was found compared to patients with de novo deletions (FSIQ 83). While previous studies have predominantly reported on the overall cognitive outcomes and the rather limited and outdated VIQ-NVIQ comparisons, detailed cognitive profiles based on primary index scores (WISC-V) remain unexplored. Additionally, longitudinal studies beyond the age of seven are lacking in the 16p11.2 population [2]. Consequently, it remains unknown how their cognitive skills continue to develop during primary and secondary school. Understanding cognitive profiles and trajectories associated with 16p11.2 CNVs has scientific value for advancing our understanding of genotype–phenotype correlations, while holding clinical importance in setting clear expectations for families and facilitating treatment planning and monitoring [2].

This study aims to address these gaps by comprehensively characterising the developmental phenotype of school-aged children with 16p11.2DS. The objective is to investigate early developmental milestones, cross-sectional cognitive profiles, and longitudinal cognitive trajectories within this population, while exploring the potential association between the attainment of developmental milestones and intelligence outcomes. Furthermore, we want to delineate the broad cognitive profiles based on the WISC-V cognitive indices to look for potential cognitive signatures of the 16p11.2DS and compare these to the existing literature based on VIQ-NVIQ comparisons. This study addresses the following research questions:

- Were early developmental milestones in language, motor and continence domains delayed in school-aged children with 16p11.2DS?
- How are the broad cognitive indices of school-aged children with 16p11.2DS characterised based on the WISC-V Primary Index Scales?
- What cognitive trajectories can be observed in school-aged children with 16p11.2 DS?
- Is the attainment of early developmental milestones associated with later intelligence outcomes in school-aged children with 16p11.2DS?

### Methodology

#### Participants

In total, 24 school-aged children between 5–16 years (median age = 10.8 years) participated in this study. Following a genetics first approach, all children had a confirmed genetic diagnosis of 16p11.2DS defined by breakpoints 4 and 5 (BP4-BP5) by microarray. Exclusion criteria included very and extreme prematurity (< 32 weeks) and the presence of additional (likely) pathogenic variants. Patient characteristics are summarised in Table 1. All patients were index patients, who were referred to the centre of Human Genetics at University Hospitals Leuven in Belgium based on developmental delays (42%, 10/24), medical concerns (4%, 1/24) or a combination of both (54%, 13/24). The deletion occurred de novo in the majority of patients (81%, 13/16), while it was inherited in 19% (3/16).

**Table 1:** Sociodemographic characteristics, educational and genetic data in 16p11.2DS (*n* = 24)

**16p11.2DS**	
Sample Size (*n*)	24
Sex (*n*, %)	
Male	10/24 (42%)
Female	14/24 (58%)
Chronological age (yrs.mo)	
Average (SD)	10.11 (3.3)
Median	10.8
Range	5.9–16.11
Inheritance pattern (*n*, %)	
De novo	13/24 (54%)
Inherited:	3/24 (13%)
- Maternally inherited	2/3 (67%)
- Paternally inherited	1/3 (33%)
Unknown*	8/24 (33%)
Socioeconomic status (*n*, %)**	
High	10/24 (42%)
Middle	12/24 (50%)
Low	2/24 (8%)
Indication for diagnosis	
Medical	1/24 (4%)
Developmental	10/24 (42%)
Medical + developmental	13/24 (54%)
Type of education (*n*, %)	
Special education	20/24 (83%)
Regular education	1/24 (4%)
Regular with assistance	3/24 (13%)
Therapy (n, %)	23/24 (96%)
Physiotherapy	18/24 (75%)
Speech-language therapy	19/24 (79%)
Occupational therapy	7/24 (29%)
Psychotherapy	3/24 (13%)
Home-based early intervention	8/24 (33%)
Formal diagnosis of autism spectrum disorder	11/23 (48%)***
IQ range (*n*, %)	
< 55	4/24 (17%)
55–70	5/24 (21%)
71–85	11/24 (46%)
86–100	4/24 (17%)

Note. *foster care (*n* = 2), maternal inheritance ruled out, parents declined genetic testing. **The educational level achieved by the (foster) mother was utilised as a substitute measure to assess socioeconomic status (SES). The categorisation of SES relied on the International Standard Classification of Education (ISCED) provided by UNESCO [31, 40]. The classification involved three main categories: low (primary education or lower high school grades), middle (secondary/high school education), and high (Bachelor's, Master's, or Doctoral Degrees). *** For one child, no data were available on formal diagnosis of autism spectrum disorder

### Procedures and measures

Patients were invited to the clinic or seen during home visits to collect data in a prospective way. Information about developmental milestones, referring to the timely achievement of milestones across several developmental areas (motor, language, continence), was gathered through clinical follow-ups or parental anamnestic reports. Data from digital medical records, in-person assessments and parental interviews were reviewed and analysed.

Following a standardised research protocol, the most recent Dutch version of the Wechsler Intelligence Scale for Children – Fifth Edition (WISC-V-NL; [14, 50] was administered in all, unless an intelligence test had been administered in the past year. This was the case for the three youngest children in our sample (5.9, 5.10 and 6.5 years), who had been tested with the Dutch versions of Snijders–Oomen Nonverbal test Revised (SON-R; [38]), Wechsler Nonverbal Scale of Ability (WNV-NL; [51]) and the Wechsler Preschool and Primary Scale of Intelligence – Fourth Edition (WPPSI-IV-NL; [49]), respectively. Using age-referenced norm tables, a Full-Scale IQ (FSIQ), five Primary Index Scales (i.e., Verbal Comprehension (VCI), Visual Spatial (VSI), Fluid Reasoning (FRI), Working Memory (WMI), and Processing Speed (PSI)) and one Ancillary Index Scale (Nonverbal (NVI)) were computed (M = 100, SD = 15) for all patients. However, for one participant with severely limited verbal abilities and two participants assessed with a nonverbal intelligence test (SON-R and WNV-NL), only NVI scores were available and used in the analyses. When evaluating disharmonic profiles based on VCI versus NVI, these three participants were excluded from the analyses.

In the majority (79%, 19/24) of patients, IQ scores were available at two or three different time points, providing insights into longitudinal cognitive trajectories. The initial evaluation involved the Dutch edition of Bayley Scales of Infant Development—Second edition (BSID-II-NL; [1, 42]), resulting in a developmental quotient (DQ), whereas for the subsequent evaluation either the Wechsler Preschool and Primary Scale of Intelligence – Third Edition (WPPSI-III-NL; [15, 48]), WPPSI-IV-NL, Wechsler Intelligence Scale for Children – Third Edition (WISC-III-NL; [47]), SON-R or WISC-V were utilised. The most recent IQ assessment was part of the current research protocol, which consisted of the WISC-V (n = 18) or the SON-R (n = 1), as described above. Intelligence scores were compared across the different time points to get insight in the longitudinal cognitive trajectories. If the non-verbal intelligence test SON-R was administered, only NVI scores were compared across time points. While different cognitive assessments were used at different ages, all IQ scores were standardised to a mean of 100 (SD = 15) using age-referenced norm tables, ensuring comparability.

Cognitive trajectories were classified into two categories: relatively stable cognitive trajectories and growing into deficit trajectories, based on a change of more than 10 IQ points between assessments [43]. A relatively stable trajectory is distinguished by the progress in raw scores on subtests, demonstrating sufficient development, while scaled and standard scores remain consistent over time. Individuals categorised as growing into deficit or experiencing a developmental lag are those who exhibit inadequate progress as they age, leading to an expanding gap in comparison to the general population. Their developmental pace is notably slower than that of their typically developing peers, resulting in reduced scaled and standard scores on specific subtests [4, 9, 36, 41, 43].

### Statistical analyses

First, we investigated whether early developmental milestones were delayed in school-aged children with 16p11.2DS. One sample Wilcoxon signed-rank t-tests were executed to explore potential age differences regarding the attainment of early motor and language milestones in the 16p11.2DS group compared to the general population, whereas Binomial tests were used to compare the proportions of participants who achieved bladder and bowel control at five years of age to the proportions in the general population. Then, cognitive profiles were investigated cross-sectionally. A one sample Student’s t-test was performed to compare IQ scores in the 16p11.2DS group to those of the general population. Bonferroni correction was applied to correct for multiple testing. One-way ANOVA was run to compare the means of the five WISC-V Primary Index Scales and the Nonverbal Index (NVI). Relative strengths and weaknesses within individuals were examined according to the WISC-V manual. In addition, the WISC-V interpretive report was used to investigate pairwise difference comparisons at the Primary Index level [14, 50]. To interpret the Index Scales results in the context of the existing literature, we also compared the Verbal Comprehension Index (VCI) and the Nonverbal Index (NVI) within individuals. Disharmonic VCI versus NVI profiles were identified as a difference of at least 15 IQ points (1 SD) between both indices [12]. Next, longitudinal cognitive trajectories were explored. With regard to longitudinal cognitive trajectories, paired Samples *t*-tests were performed to compare IQ outcomes between the first and second, and second and third time point. Cohen’s *d* was used as effect size parameter with values of ≥0.2, ≥0.5, ≥0.8 interpreted as small, moderate or large effects respectively. Finally, Pearson product-moment correlation analyses were run to investigate the relation between the achievement of early developmental milestones and IQ outcomes. Statistical analyses were run using JASP [18] and R [33, 53].

## Results

### Attainment of developmental milestones in 16p11.2DS

**Fig. 1:**
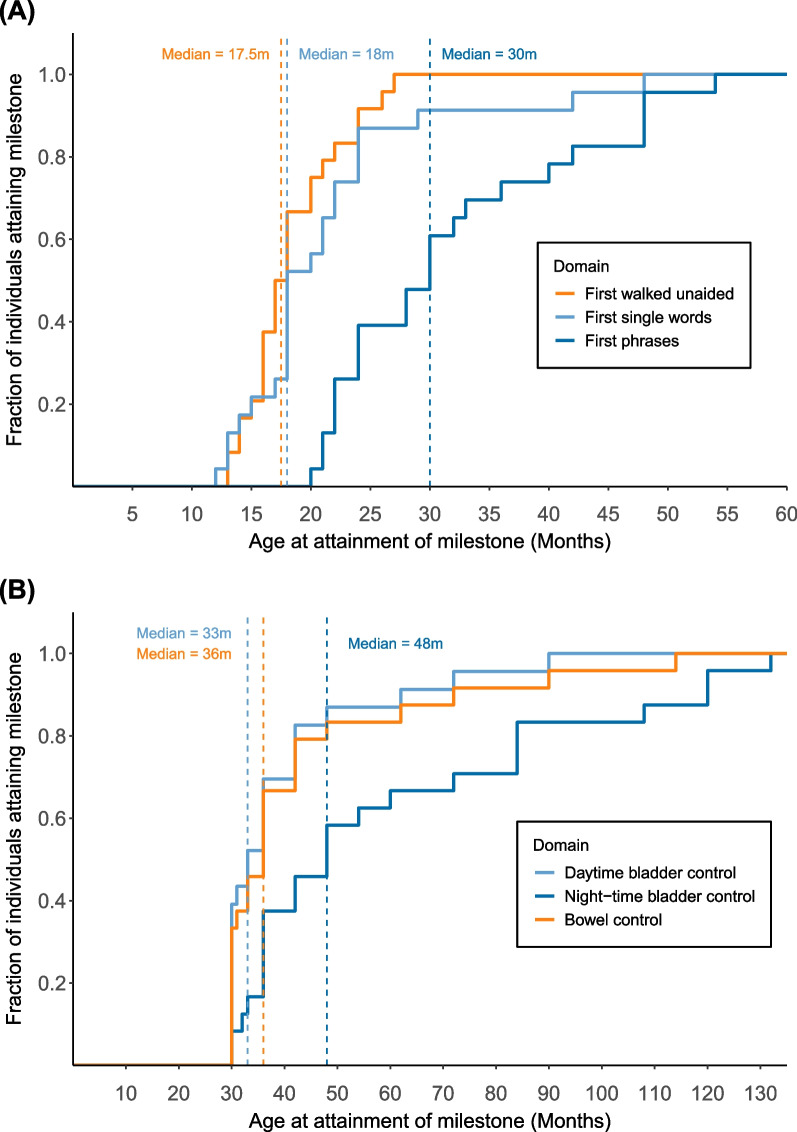
Developmental trajectory of participants with 16p11.2DS. (A) Age at attainment of gross motor and language milestones. The solid lines indicate the time course of achievement of walking independently or speaking the first words/phrases by our cohort. (B) Age at attainment of continence milestones. The solid lines indicate the time course of achievement of day- and night-time bladder and bowel control by our cohort. The dashed lines show the median age at the attainment of these milestones in our cohort

Figure 1 depicts the proportion of children attaining developmental milestones across several domains as a function of age, reflecting a wide age range. The dotted lines represent the median age of milestone achievement in the 16p11.2DS group. Children with 16p11.2DS first walked independently at a median age of 17.5 months, while their first words and simple phrases were spoken at a median age of 18 and 30 months respectively. None of the children with 16p11.2DS could walk independently at 12 months, which is the mean age in the typical population [52], and only one of the children used a single word by that age [7, 34, 44]. Twenty-six percent (6/23) formed simple phrases by combining two words at the mean age of the normative population (24 months). Daytime bladder control and bowel control were attained at median ages of 33 and 36 months respectively, whereas night-time bladder control was achieved at a median age of 4 years (48 months).

Table 2 provides the descriptive statistics of the attainment of developmental milestones in children with 16p11.2DS. One sample Wilcoxon-signed rank t-tests were used to compare the milestones to these of typically developing children in the general population. The results show statistically significant delays in the attainment of both language and motor milestones among children with 16p11.2DS, with large effect sizes. Furthermore, using binomial tests, we found no statistically significant difference in the proportion of children with diurnal enuresis compared to the general population (13%/17% vs. 7%). However, a significantly higher proportion of individuals with 16p11.2DS experienced nocturnal enuresis at age 5 (60 months) compared to the proportion in the general population (38% vs. 17%).

**Table 2:** Developmental milestones and FSIQ in 16p11.2DS (n = 24) compared to the general population

**Developmental milestones and FSIQ**	**16p11.2DS** M (SD) Median Range	**Statistical outcomes** **group level** One sample t-test (t/*V* =, *p* = *,d/r* =*)* *Or Binomial test* *(p* =*)*
First walked unaided (months)		
Average (SD)	18 (4)	*V* = 300.000
Median	17.5	*p* < 0.001**
Range	13–27	*r* = 1.000
Motor delays (*n*, %)	16/24 (67%)	
Age of first single words (months)		
Average (SD)	21 (9)	*V* = 253.000
Median	18	*p* < 0.001**
Range	12–48	*r* = 1.000
Age of first single phrases (months)		
Average (SD)	32 (10)	*V* = 188.000
Median	30	*p* = 0.002**
Range	20–54	*r* = 0.790
Speech-language delays (*n*, %)	22/24 (92%)	
Age at daytime bladder control (months)		
Average (SD)	40 (15)	*p* = 0.215
Median	33	
Range	30–90	
Bladder control delays (*n, %*)	3/24 (13%)	
Age at night-time bladder control (months)		
Average (SD)	60 (32)	*p* = 0.013**
Median	48	
Range	30–132	
Bladder control delays (*n, %*)	9/24 (38%)	
Age of bowel control (months)		
Average (SD)	43 (21)	*p* = 0.083
Median	36	
Range	30–114	
Bowel control delays (*n, %*)	4/24 (17%)	
FSIQ		
Average (SD)	71 (13)	*t* = −10.466
Median	74	*p* < 0.001**
Range	45–91	*d* = −2.284
Intellectual disability (*n, %*)	9/24 (38%)	

Note. *significant at *p* < 0.05, **significant after Bonferroni correction at *p* < 0.016. Abbreviations: IQ, intellectual quotient; SD, standard deviation. First walked unaided/first single words (norm group average = 12 months, cut-off > 18 months = delayed), First single phrases (norm group average = 24 months, > 30 months = delayed), Daytime bladder/bowel control (no control in 7% at 60 months in norm group), night-time bladder control (no control in 17% at 60 months in norm group), FSIQ (norm group average = 100, cut-off < 70 = intellectual disability) [7, 21, 30, 34, 44, 52]

### Cross-sectional cognitive profiles in 16p11.2DS

**Fig. 2:**
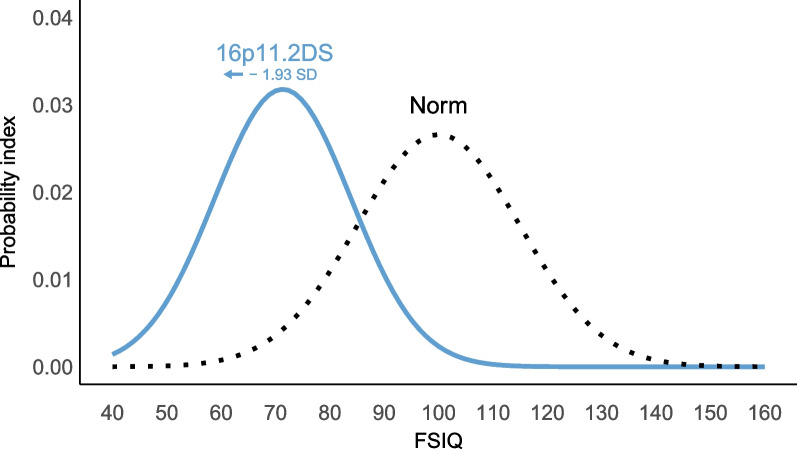
Gaussian curve of FSIQ in 16p11.2DS (n = 21). The dashed line depicts the normal distribution of FSIQ in the general population (M = 100, SD = 15). SD shifts are calculated in relation to the norm group sample

The average FSIQ in our cohort at the most recent timepoint is 71, with 38% exhibiting mild-to-moderate ID. Figure 2 illustrates that the gaussian curve of the 16p11.2DS group has shifted approximately 30 IQ points (≈ 1.93SD) to the left, in comparison to the distribution of the general population (M = 100, SD = 15) . A one sample Student’s *t*-test reported in Table 2 confirmed statistically significantly lower FSIQ scores in school-aged children with 16p11.2DS compared to their typically developing peers in the general population, with a large effect size. Descriptive statistics and boxplots on potentially confounding factors, such as inheritance pattern, comorbid ASD or attention-deficit/hyperactivity disorder (ADHD), and sex, can be found in Supplementary Table 1 and Supplementary Fig. 1.

**Fig. 3:**
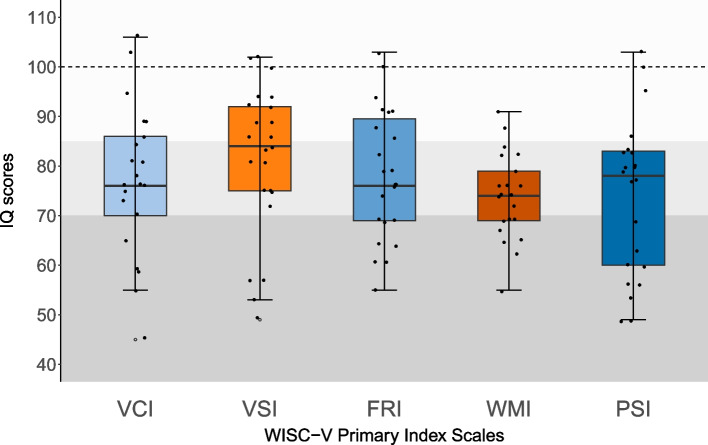
Boxplots across WISC-V Primary Index Scales in 16p11.2DS. The dashed line represents the norm group average (M = 100, SD = 15). The grey zones delineate borderline IQ (70–84) and mild-moderate IQ (< 70). Abbreviations: VCI, Verbal Comprehension Index; VSI, Visual Spatial Index; FRI, Fluid Reasoning Index; WMI, Working Memory Index; PSI, Processing Speed Index

Figure 3 displays boxplots of the IQ scores of the five WISC-V Primary Index Scales. The grey zones delineate the areas of borderline IQ (IQ 70–84) and mild-moderate ID (< 70), whereas the dashed line depicts the norm group average (M = 100). The average scores of the five Primary Index Scales are in the borderline range (IQ 70–84, Supplementary Table 2). One-way ANOVA revealed no statistically significant differences across the five Primary Index Scales and Nonverbal Index (*F*(5) = 1.287, *p* = 0.274, *η*^2^ = 0.049).

Supplementary Fig. 2 shows the individual Index profiles for all children with 16p11.2DS, demonstrating both intra- and interindividual variability across the five Primary Indices. Examining relative strengths and weaknesses within individuals according to the WISC-V manual revealed that the Visual Spatial index is the index for which most individuals exhibit a relative strength (35%, 7/20) and none a relative weakness. Using the WISC-V interpretive report, pairwise difference comparisons at the Primary Index level revealed that all individuals (19/19) demonstrated at least one (1/10) significant difference, with 79% (15/19) showing at least three (3/10) significant differences (Supplementary Table 3). To interpret the Index Scales results in the context of the existing literature, we also compared the Verbal Comprehension Index (VCI) and the Nonverbal Index (NVI) within individuals. Disharmonic VCI versus NVI profiles were identified as a difference of at least 15 IQ points (1 SD) between both indices [12] and observed in 55% (11/20) of children, with 40% (8/20) showing significantly higher VCI, whereas 15% (3/20) demonstrated significantly higher NVI. More details on the Primary Index Scales can be found in Supplementary Table 2.

### Longitudinal cognitive trajectories in 16p11.2DS

A subgroup of individuals had formal IQ assessment at two or three time points. The comparison in the youngest group (n = 11) included a first evaluation by the BSID-II-NL in early toddlerhood at a median age of 30 months (2.6 years, age range 1.3–3.2 years) and a second evaluation by the SON-R or WPPSI-III in preschool at a median age of 5.7 years (age range 3.7–6.9 years). The mean change in IQ score between these two measurements showed a 12-point increase in favour of the second assessment (see Supplementary Fig. 4). A Paired Samples *t-*test within participants revealed statistically significantly lower scores (t(10) = -2.862, p = 0.017, d = -0.863) at the first time point (average developmental quotient T1 = 71, range 55–91) than at the second time point (average IQ T2 = 83, range 50 –105), with a large effect size.

**Fig. 4:**
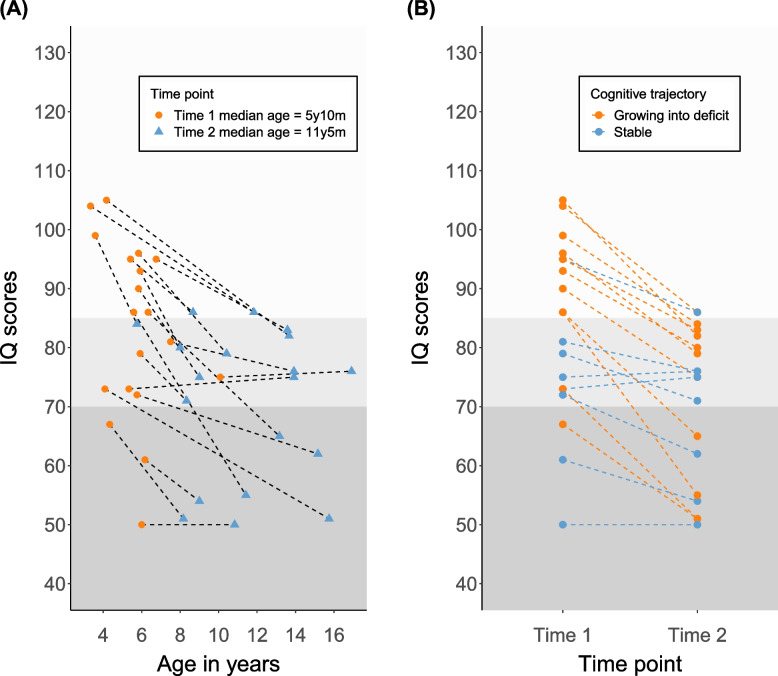
Longitudinal cognitive trajectories in children with 16p11.2DS (n = 19). (A) IQ scores as a function of age for each participant. The colour and shape refer to the time point. The dashed lines represent the individual cognitive trajectories. (B) IQ scores at two different time points (median age T1 5.10 years, median age T2 11.5 years). The colours refer to the cognitive trajectories: stable (|IQ T2 – T1| < 10) and growing into deficit (IQ T1 – T2 >10)

The comparison in the oldest group (n = 19) consisted of a first evaluation by the SON-R, WPPSI-III or WISC-III at a median age of 5.10 years (age range 3.4 years – 10.1 years) and a second evaluation by the WISC-V or SON-R at a median age of 11.5 years (age range 5.9–16.11 years). The IQ scores at both time points are plotted in Fig. 4. The dotted lines indicate the cognitive trajectory of each participant, revealing a downward trend towards the second time point. A Paired Samples *t*-test confirmed statistically significantly lower IQ scores with large effect size at the second and most recent time point (average IQ T1 = 83, average IQ T2 = 71, *t*(18) = -6.297, *p* < 0.001, *d* = -1.445). Forty-two percent (8/19) of children with 16p11.2DS demonstrated a relatively stable cognitive trajectory, whereas 58% (11/19) showed a growing into deficit trajectory, indicated by the difference of more than 10 IQ points between the two assessments.

### Association between developmental milestones and FSIQ scores in 16p11.2DS

Pearson correlation analyses were run to investigate the potential relation between the age at attainment of early developmental milestones across motor, language and continence domains and IQ scores in the 16p11.2DS population. Correlation plots can be found in Supplementary Fig. 3. None of the developmental milestones were statistically significantly associated with the FSIQ outcomes in the 16p11.2DS group (*r* < 0.271, 0.235 > *p* > 0.913). .

## Discussion

The aim of the current study was to characterise developmental milestones, broad cognitive indices and cognitive trajectories of 24 school-aged children with the proximal BP4-BP5 16p11.2DS, using a standardised protocol that consisted of in-person formal cognitive assessments, parental interviews and reviewing digital medical records.

Regarding developmental milestones, results indicated that almost all children with 16p11.2DS (92%) experienced language delays, whereas 67% showed motor delays. Mean age of walking was at 18 months of age, which was similar to the mean age found by Zufferey et al. [54]. Compared to the general population, median ages of achieving motor and language milestones were significantly delayed. In addition, significantly more children with 16p11.2DS experienced nocturnal enuresis at 60 months of age. In general, incontinence is more often reported in children with genetic syndromes and/or neurodevelopmental disorders such as ID and autism spectrum disorders [24, 45]. Moreover, within these syndromes, the prevalence of incontinence increases in children with more severe levels of ID. However, in the current study, we did not find any significant associations between bladder or bowel control and IQ. Possible factors that might play a role in the delayed age of reaching night-time bladder control in genetic populations could be the presence of epilepsy and/or motor problems [45], which are common in 16p11.2DS, but this warrants further research. Another older study in a large sample (n = 1666) of typically developing children concluded that nocturnal enuresis might be associated with motor and language milestones [39]. However, we could not confirm these associations in the present study, which might be partially due to the much smaller sample.

The average FSIQ of children with 16p11.2DS in the current study is 71, whereas other studies have reported average FSIQ scores ranging from 69 to 92 [3, 8, 13, 19, 20, 22, 25–27]. Consequently, the current cohort is situated at the lower end of the IQ-spectrum, which might be partially explained by methodological differences, such as used test instruments, age differences, and distinct ascertainment strategies dependent on the clinical setting. In addition, 38% of our cohort showed mild-to-moderate ID, aligning with findings by Niarchou et al. [29], who reported ID in a similar proportion (30%) of patients.

To the best of our knowledge, the current study is the first to examine the five WISC-V Primary Index Scales, revealing both intra- and interindividual variability across the five broad cognitive indices. At the group level, all mean Index IQ scores (Verbal Comprehension, Perceptual Reasoning, Fluid Reasoning, Working Memory and Processing Speed) fell within the borderline range, with no statistically significant differences across indices. However, individual-level analyses showed that cognitive profiles were highly variable as the majority of the sample (79%) exhibited at least three clinically relevant differences between Index scores. A notable trend was that Visual Spatial skills emerged as a relative strength in 35% of children. In addition, 55% (11/20) showed a disharmonic VCI-NVI profile, with 40% (8/20) performing better in verbal skills. However, the proportion of children with stronger nonverbal skills may be underestimated, as two children were assessed using a nonverbal test and one had insufficient verbal abilities; therefore, they were not included in these analyses.

These findings contrast with previous studies that suggested a greater impact on verbal than nonverbal skills [3, 8, 13, 16, 20, 27, 28, 32, 54], with significant differences only observed in Zufferey et al. [54], although many of these studies relied on overlapping cohorts. Bernier et al. [2], however, reported, stronger verbal skills from age 5 onward, aligning more closely with the current results. Differences in cognitive profiles across studies may stem from differences in IQ measurements and subtests used to assess verbal and nonverbal skills. Future studies should further investigate these Index Scales to fully delineate the broad intellectual and cognitive profile of children with 16p11.2DS.

Both the lack of group-level differences and the substantial inter-individual variability underscore the importance of not only considering group averages but also carefully assessing each child's individual performance. On the other hand, the substantial intra-individual variability points to the importance of looking beyond FSIQ outcomes. Analysing the different Index Scales of the WISC V-NL enables us to gain a comprehensive understanding of an individual’s broader cognitive capacities, and it allows us to tailor interventions accordingly to the specific strengths and weaknesses.

Longitudinal cognitive trajectories in the youngest group revealed a significant 12-point increase from the first assessment in early toddlerhood to the second assessment in preschool, partially corresponding to the results from Bernier et al. [2], who observed improvements in verbal IQ from 2 to 7 years of age. However, it is essential to acknowledge the limitations of the developmental quotient (DQ) derived from the BSID-II. One study reported that the DQ of the BSID-II is an insufficient predictor of later IQ in typically developing children [23]. Furthermore, it is important to recognise that the DQ of BSID-II is not entirely equivalent to an IQ-score, as it encompasses a broader range of abilities, including cognitive, language and motor skills. Since language and motor delays are common in the current group of children, these factors might have contributed to the initially overall lower DQ in early toddlerhood. In addition, test-taking behaviour could have influenced score improvements over time, as older children may perform better due to increased motivation, attention, and engagement. Many children in this study also received speech-language or physiotherapy during early toddlerhood, suggesting that the observed increase towards the second assessment may, in part, reflect positive responses to therapeutic interventions. An alternative hypothesis is that at this early stage, predominantly children with more pronounced developmental delays are referred for formal DQ assessment, and therefore primarily represent the more severe end of the spectrum. Given the small sample size, the current results should be interpreted with caution.

Longitudinal cognitive trajectories in the oldest age group revealed a significant 13-point decrease from the first assessment in preschool to the second assessment in primary or the beginning of secondary school. Growing into deficit-trajectories were identified in 58% (11/19) of children. These results should be approached with some caution, due to the limited sample size, the use of different IQ measurements, and partially overlapping age ranges at the two time points. Nevertheless, growing into deficit-profiles have also been observed in other recurrent CNV populations, such as 22q11.2 deletion syndrome or 22q11.2 duplication [4, 9, 36, 41, 43]. These profiles might be partly accounted for by the rising emphasis on abstract reasoning skills in IQ measurements as children age, which could represent a relative weakness in 16p11.2DS. These longitudinal cognitive trajectories underscore the importance of regular cognitive (follow-up) assessments using standardised measures to provide individualised and adapted/adequate support as early as possible, since challenges may change at different ages and life stages.

### Strengths, limitations and future

A key strength of the current study is the use of a standardised protocol for the prospective collection of cognitive and behavioural data, incorporating gold standard test instruments to assess IQ in person. This robust methodology is complemented by data collected from digital medical records and parental interviews on developmental history. In addition, the focus on a pediatric sample (5–16 years) contributes to a clearer understanding of the developmental and cognitive phenotype among primary and early secondary school-aged children with 16p11.2DS.

Despite these strengths, certain limitations should be acknowledged. The relatively small sample size and the potential ascertainment bias within the current cohort prevent us from drawing generalised conclusions. Since all participants were probands diagnosed due to developmental or medical concerns, the cohort may not fully represent the broader phenotypic spectrum of 16p11.2DS, particularly milder cases identified through familial screening. Future studies in larger cohorts both from clinical and research settings, including carrier relatives, are needed to gain more insight into the penetrance and phenotypic variability of 16p11.2DS. Moreover, the use of different IQ tests, different ages at previous IQ assessments and the lack of more detailed data on the index scores poses a challenge for cross-time point comparisons. Longitudinal studies should be performed with the same IQ tests to assess IQ consistently at different time points and extend into adulthood. Additionally, future research should preferably include assessments of adaptive skills, since these are crucial for our understanding of the cognitive and functional outcomes in individuals with 16p11.2DS.

## Conclusion

The present study aimed to elucidate developmental milestones, cross-sectional cognitive profiles and longitudinal cognitive trajectories of school-aged children with the proximal BP4-BP5 16p11.2DS. Our findings reveal a high prevalence of delayed motor, language and night-time bladder control milestones in individuals with 16p11.2DS, independent of their later cognitive outcomes. This is an important clinical observation that paediatricians and other healthcare professionals should be aware of. Children in early toddlerhood already demonstrate diverse cognitive profiles, with a subgroup undergoing formal IQ assessment due to pronounced developmental, motor and language delays. Most of them show positive responses to therapy, demonstrating the importance of early interventions. School-aged children with 16p11.2DS show increasing cognitive impairments with age, underscoring the need for regular cognitive assessments, follow-up and personalised educational intervention strategies. The large intra-individual variability shown by the high proportion of disharmonic cognitive profiles emphasises the need not only to look at FSIQ but also at the complete profile of cognitive domains including Verbal Comprehension, Perceptual Reasoning, Fluid Reasoning, Working Memory and Processing Speed. Finally, to fully capture and deepen our understanding of the penetrance and phenotypic variability of development and cognitive outcomes in 16p11.2DS, future studies should encompass larger cohorts, including carrier relatives.

## Supplementary Information

Additional file 1.

Additional file 2: Supplementary Table 1. Descriptive statistics WISC-V FSIQ scores across subgroups based on potential confounding factors

Additional file 3: Supplementary Table 2. Descriptive statistics WISC-V Primary Index Scales and one Ancillary Index Scale

Additional file 4: Supplementary Table 3. Counts of Index level pairwise difference comparisons

Additional file 5: Supplementary Figure 1. Boxplots FSIQ scores dependent on potential confounding factors inheritance pattern, presence of a formal ASD or ADHD diagnosis and sex

Additional file 6: Supplementary Figure 2. WISC-V Primary Index Scales across patients

Additional file 7: Supplementary Figure 3. Scatterplots FSIQ and early developmental milestones

Additional file 8: Supplementary Figure 4. Longitudinal cognitive trajectories in youngest comparison group of children with 16p11.2DS (*n*=11)

## Data Availability

The datasets generated and/or analysed during the current study are available from the corresponding author on reasonable request.
