# Supplementary material for: Developmental milestones and cognitive trajectories in school-aged children with 16p11.2 deletion
Source: J Neurodev Disord. 2025 Jun 19;17:33. doi: 10.1186/s11689-025-09615-7 (PMC12178036; doi:10.1186/s11689-025-09615-7)
Supplement: Supplementary file 1 — Additional file 1. [file 11689_2025_9615_MOESM1_ESM.docx]

Descriptive statistics and boxplots on potentially confounding factors, such as inheritance pattern, comorbid ASD or attention-deficit/hyperactivity disorder (ADHD), and sex, can be found in supplementary Table 1 and supplementary Figure 1. Qualitatively, FSIQ scores of children with de novo deletions $\left( n=10, M=73 \right)$ are 10 points higher than those of children with inherited deletions $(n=3,M=63)$. Children with ASD $\left( n=9, M=70 \right)$ performed on average similar to children without ASD $(n=11, M=72)$. Children with ADHD $(n=7,M=66)$ obtained on average lower FSIQ scores compared to children without ADHD $(n=14, M=74)$. In addition, FSIQ outcomes of boys $\left( n=8,M=77 \right)$ were on average 9 points higher than those of girls $(n=13,M=68)$. Age did not have an influence on the FSIQ outcomes $(p=0.502)$.

Qualitatively, children with de novo 16p11.2DS achieved higher FSIQ compared to those with inherited 16p11.2DS, consistent with observations by Zufferey et al. (2012). It is noteworthy that FSIQ scores did not exhibit large or clinically relevant differences among children with comorbid neurodevelopmental disorders, neither were these scores dependent on sex or age. However, these preliminary, descriptive findings should be validated in a larger sample.

Table 1 – Descriptive statistiscs WISC-V FSIQ scores across subgroups based on potential confounding factors

| **Inheritance pattern** | De novo  N  Average (SD)  Median  Range | 3  63 (11)  68  51 – 71 |
| --- | --- | --- |
|  | Inherited  N  Average (SD)  Median  Range | 10  73 (13)  76  45 – 89 |
| **ASD diagnosis** | ASD  N  Average (SD)  Median  Range | 9  70 (11)  74  51 – 83 |
|  | No ASD  N  Average (SD)  Median  Range | 11  72 (15)  73  45 – 91 |
| **ADHD diagnosis** | ADHD  N  Average (SD)  Median  Range | 7  66 (15)  65  45 – 86 |
|  | No ADHD  N  Average (SD)  Median  Range | 14  74 (11)  76  54 – 91 |
| **Sex** | Female  N  Average (SD)  Median  Range | 13  68 (14)  71  45 – 89 |
|  | Male  N  Average (SD)  Median  Range | 8  77 (9)  75  65 – 91 |

Table 2 – Descriptive statistics WISC-V Primary Index Scales and one Ancillary Index Scale

| **WISC-V five Primary Index Scales** | Verbal Comprehension Index (VCI)  N  Average (SD)  Median  Range | 21  77 (15)  76  45 – 106 |
| --- | --- | --- |
|  | Visual Spatial Index (VSI)  N  Average (SD)  Median  Range | 23  81 (15)  84  49 – 102 |
|  | Fluid Reasoning Index (FRI)  N  Average (SD)  Median  Range | 23  78 (13)  76  55 – 103 |
|  | Working Memory Index (WMI)  N  Average (SD)  Median  Range | 21  74 (9)  74  55 – 91 |
|  | Processing Speed Index (PSI)  N  Average (SD)  Median  Range | 22  74 (16)  78  49 – 103 |
| **WISC-V Ancillary Index Scale** | Nonverbal Index (NVI)  N  Average (SD)  Median  Range | 23  73 (14)  75  50 – 94 |

Table 3 – Counts of Index level pairwise difference comparisons

| **Counts of Index level pairwise difference comparisons (out of 10)** | **Frequency** | **Proportion** |
| --- | --- | --- |
| 1 significant pairwise difference | 3/19 | 16% |
| 2 significant pairwise differences | 1/19 | 5% |
| 3 significant pairwise differences | 3/19 | 16% |
| 4 significant pairwise differences | 2/19 | 10% |
| 5 significant pairwise differences | 6/19 | 32% |
| 6 significant pairwise differences | 3/19 | 16% |
| 7 significant pairwise differences | 1/19 | 5% |


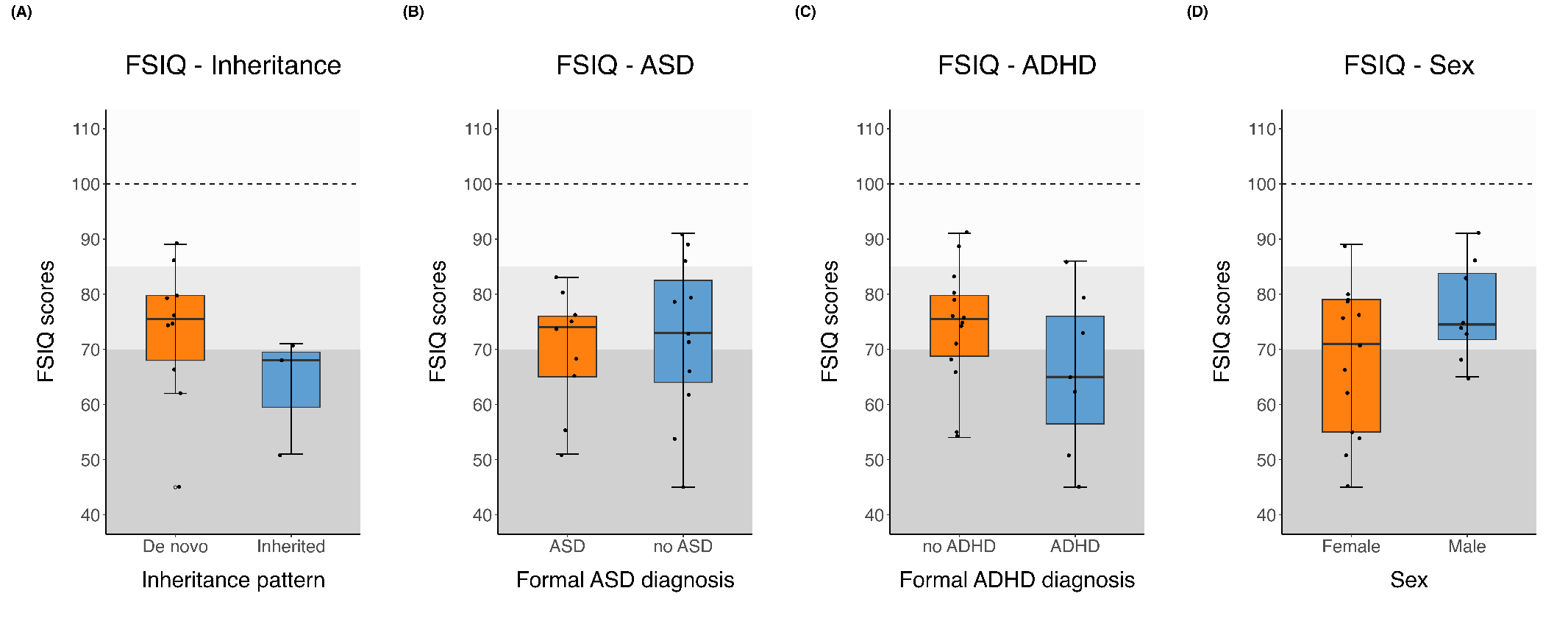


Figure 1 – Boxplots FSIQ scores dependent on potential confounding factors inheritance pattern, presence of a formal ASD or ADHD diagnosis and sex.


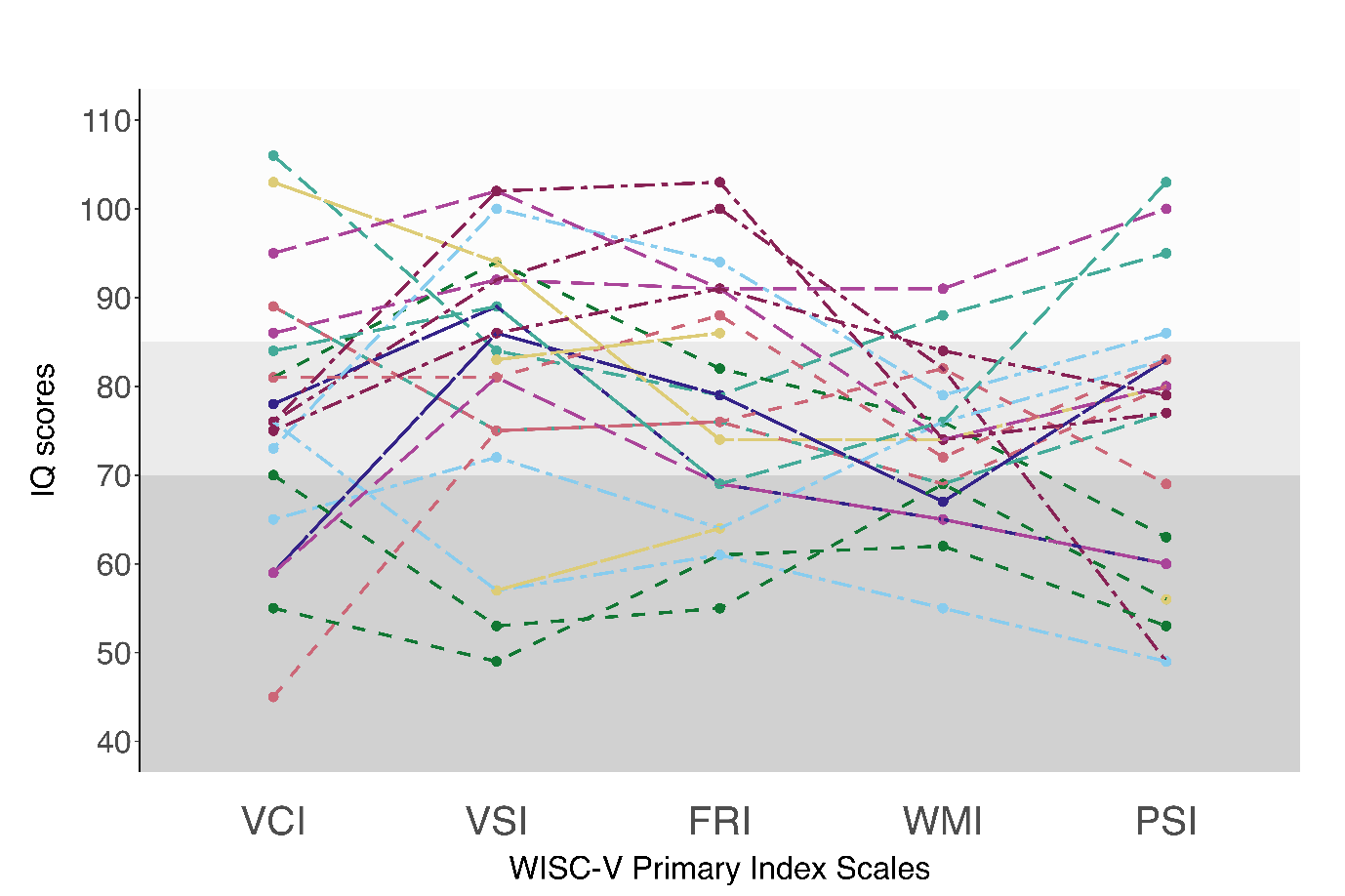


Figure 2 – WISC-V Primary Index Scales across patients. The individual lines represent the individual patient profiles across the five WISC-V Primary Index Scales. The grey zones delineate borderline IQ (70-84) and mild-moderate IQ (<70). Abbreviations: VCI, Verbal Comprehension Index; VSI, Visual Spatial Index; FRI, Fluid Reasoning Index; WMI, Working Memory Index; PSI, Processing Speed Index.


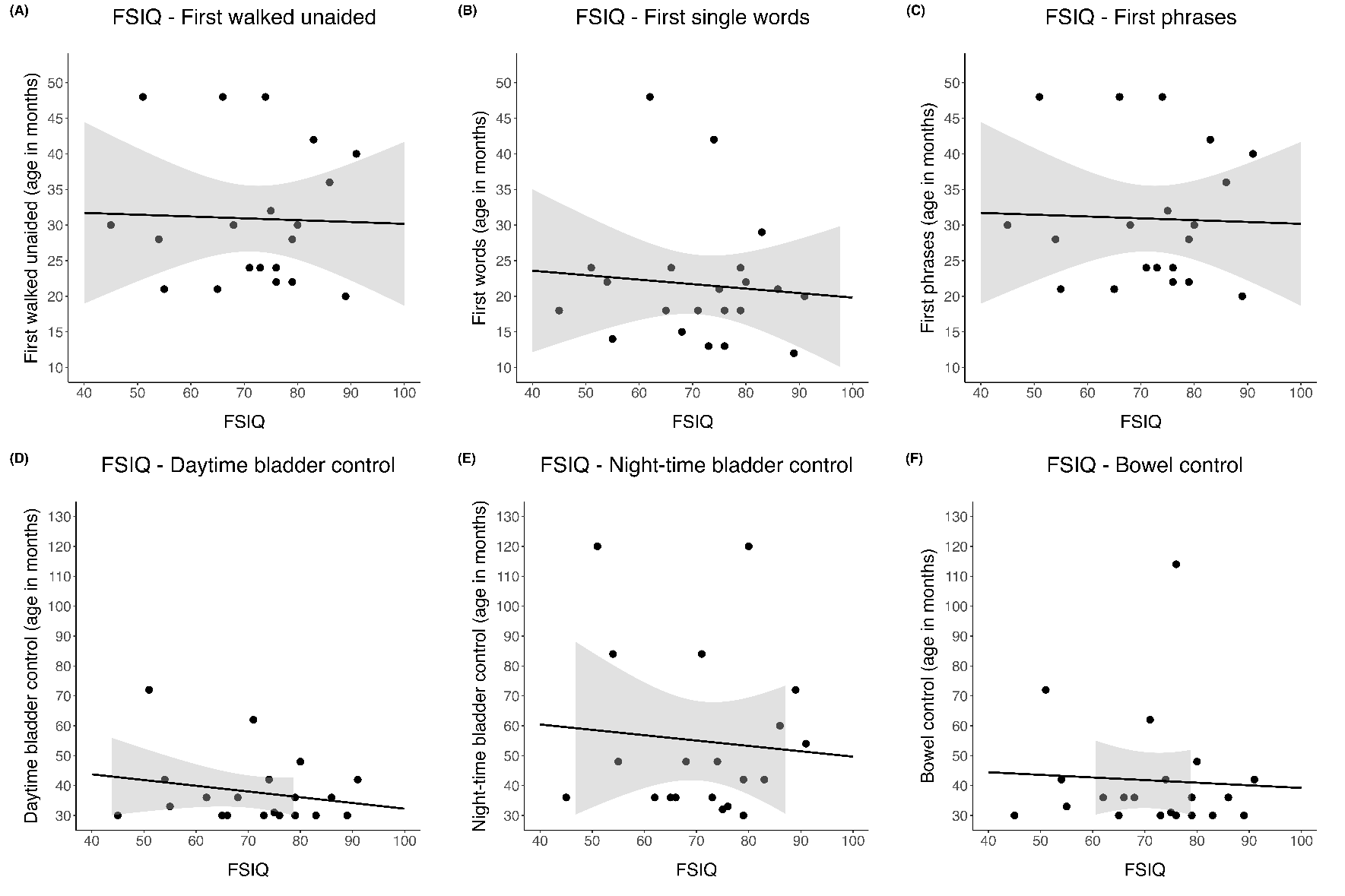


Figure 3 – Scatterplots FSIQ and early developmental milestones. (A) Association FSIQ and First walked unaided $(r=-0.271, p=0.235)$; (B) Association FSIQ and First single words $\left( r=-0.088,p=0.703 \right)$; (C) Association FSIQ and First phrases (at least two words) $\left( r=-0.110,p=0.634 \right)$; (D) Association FSIQ and Daytime bladder control $\left( r=-0.215,p=0.362 \right)$; (E) Association FSIQ and Night-time bladder control $(r=-0.025, p=0.913)$; (F) Association FSIQ and Bowel control $(r=-0.055,p=0.812)$.


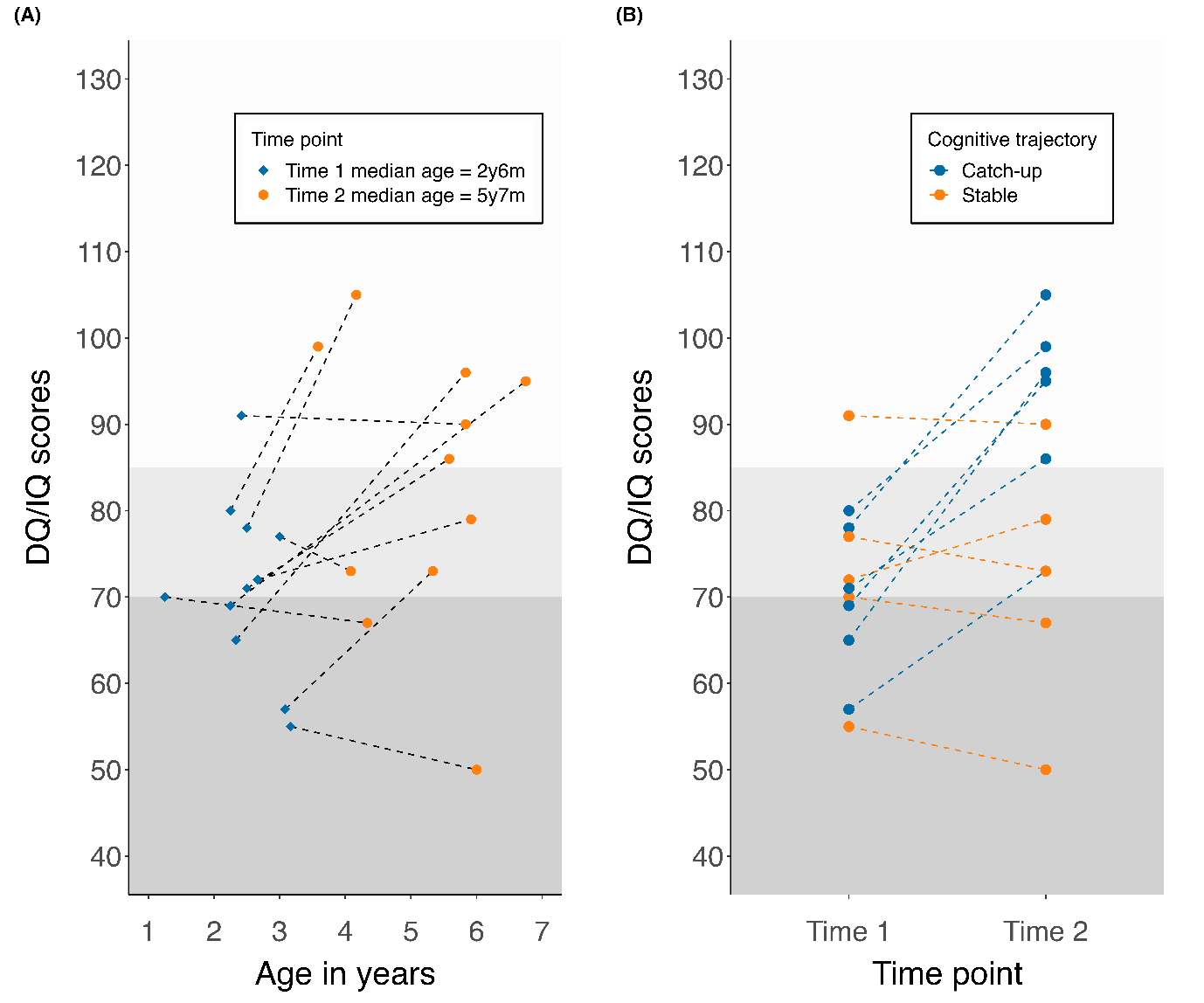


Figure 4 – Longitudinal cognitive trajectories in youngest comparison group of children with 16p11.2DS (n=11). (A) IQ scores as a function of age for each participant. The colour and shape refer to the time point. The dashed lines represent the individual cognitive trajectories. (B) IQ scores at two different time points (median age T1 2y6m, median age T2 5y7m). The colours refer to the cognitive trajectories: stable $(\left| IQ T2-T1 \right|<10)$ and catch-up $(IQ T2-T1>10)$. In total, 6/11 (55%) children caught-up with peers, whereas 5/11 (45%) showed a relatively stable cognitive profile. Abbreviations: DQ, developmental quotient; IQ, intellectual quotient.
