# Supplementary figures and images for: Developmental milestones and cognitive trajectories in school-aged children with 16p11.2 deletion
Source: J Neurodev Disord. 2025 Jun 19;17:33. doi: 10.1186/s11689-025-09615-7 (PMC12178036; doi:10.1186/s11689-025-09615-7)

**(A)****FSIQ – Inheritance**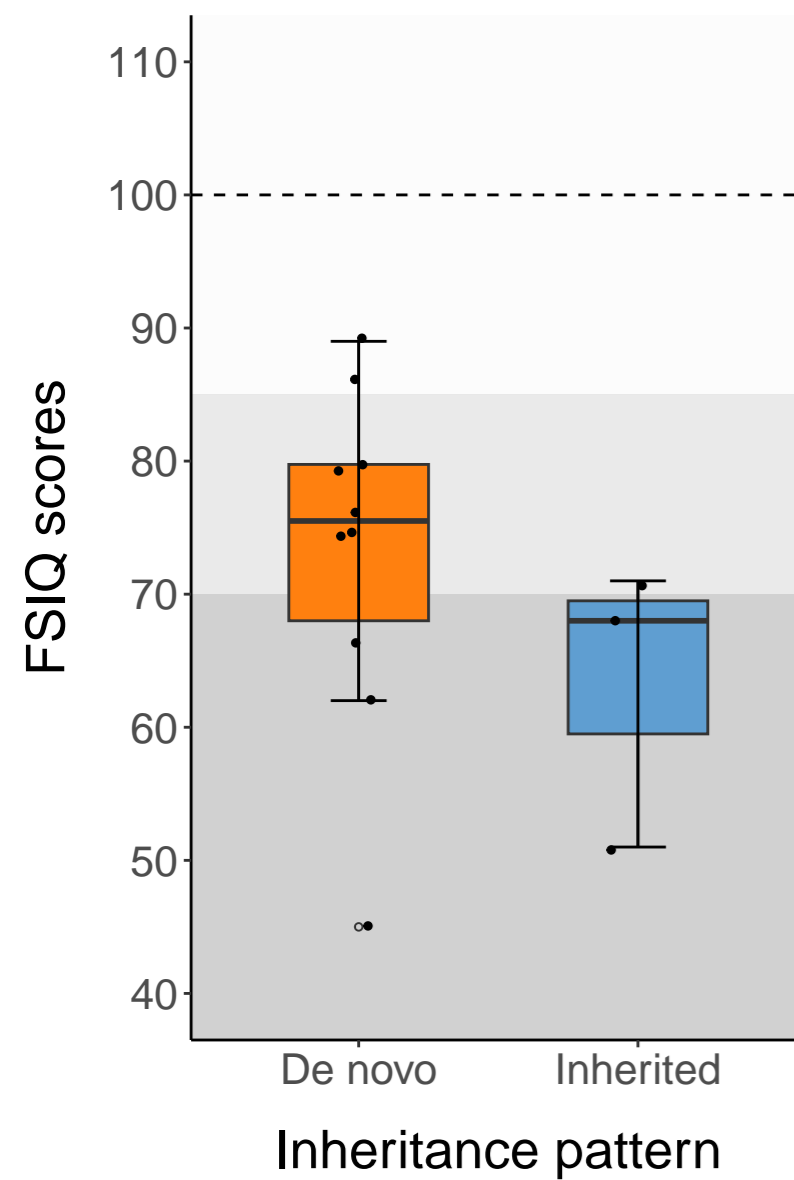**(B)****FSIQ – ASD**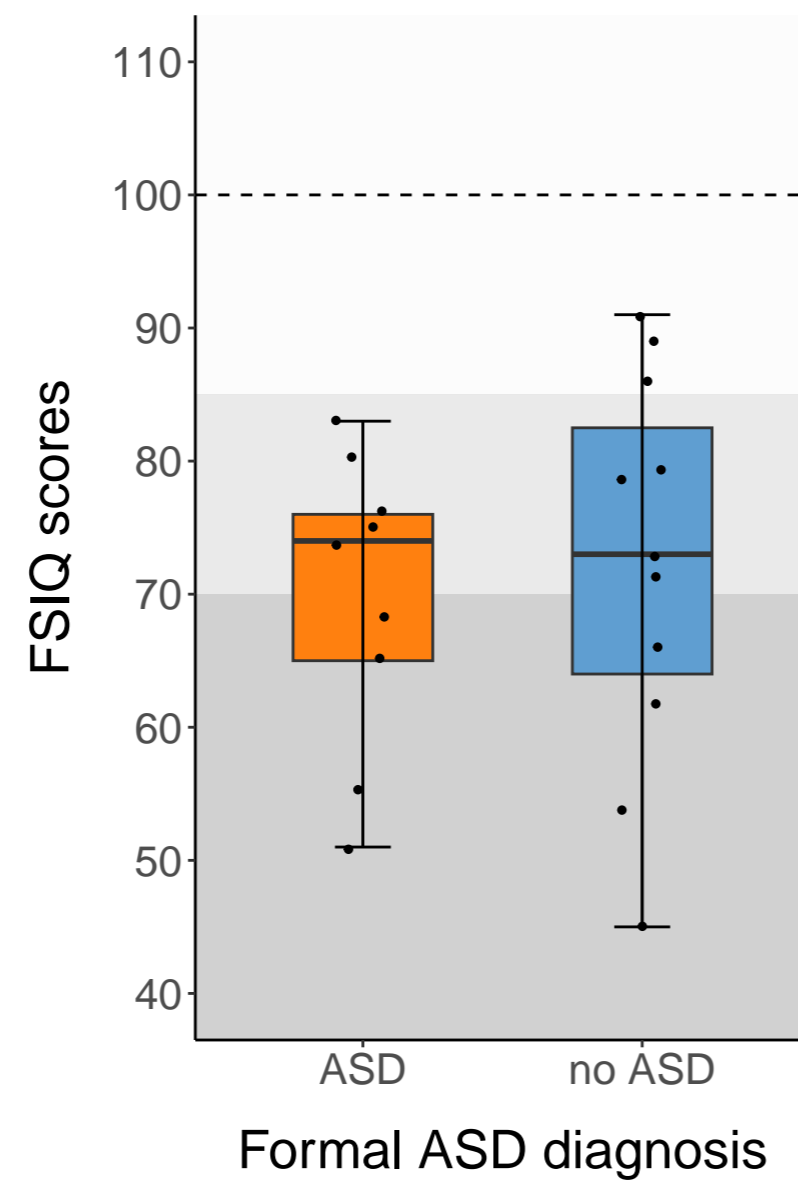**(C)****FSIQ – ADHD**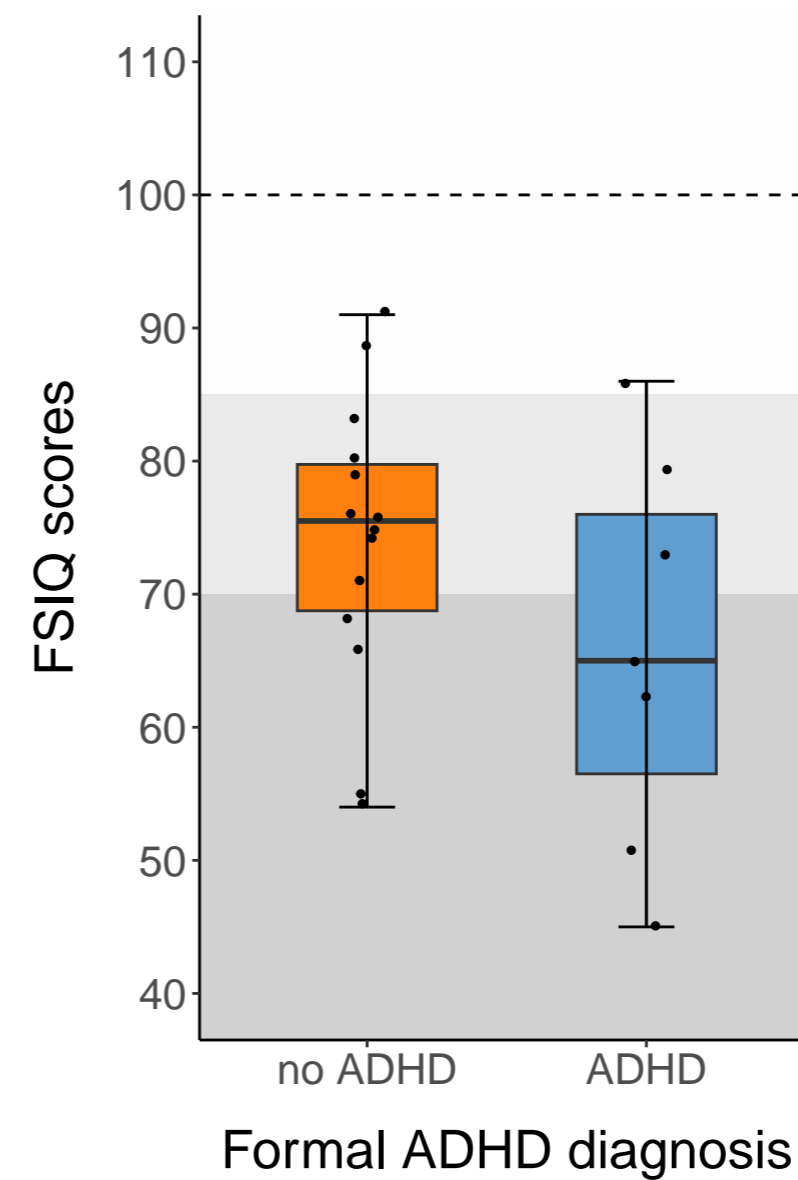**(D)****FSIQ – Sex**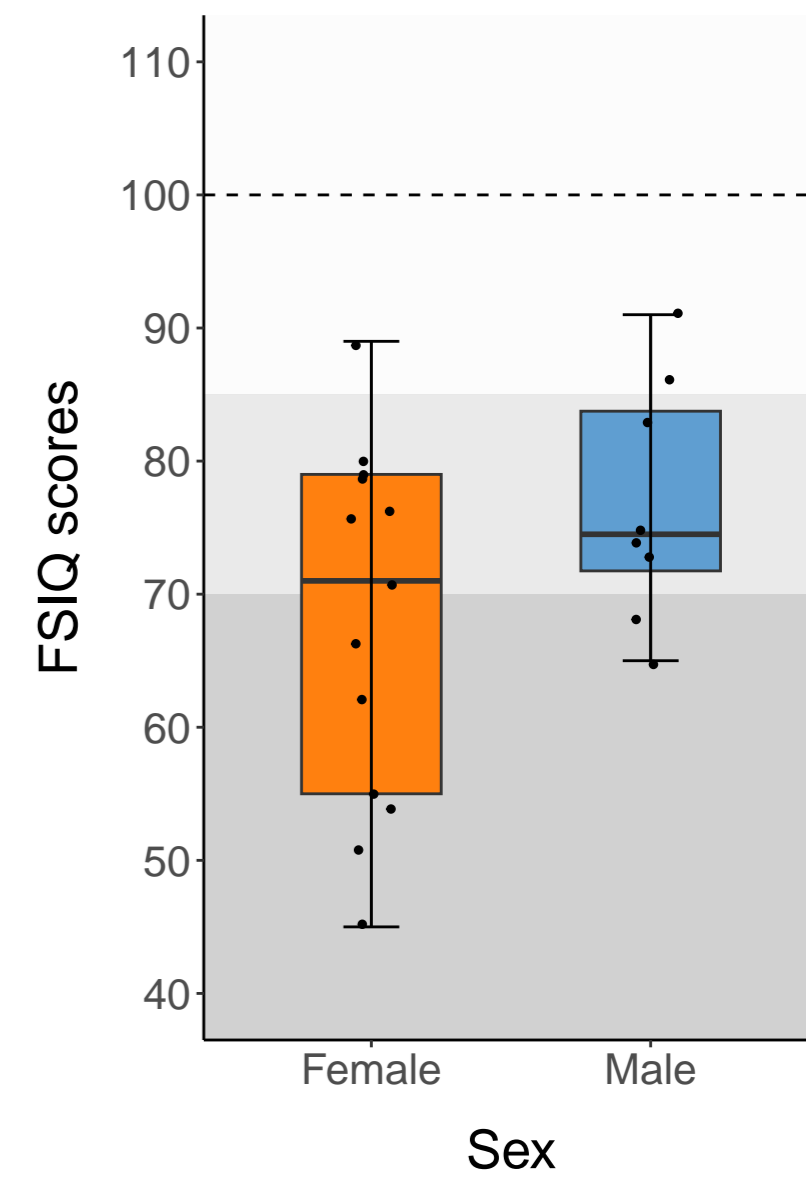

Supplement: Supplementary file 5 — Additional file 5: Supplementary Figure 1. Boxplots FSIQ scores dependent on potential confounding factors inheritance pattern, presence of a formal ASD or ADHD diagnosis and sex [file 11689_2025_9615_MOESM5_ESM.zip › Verbesselt_Supplementary_Figure 1.pdf]

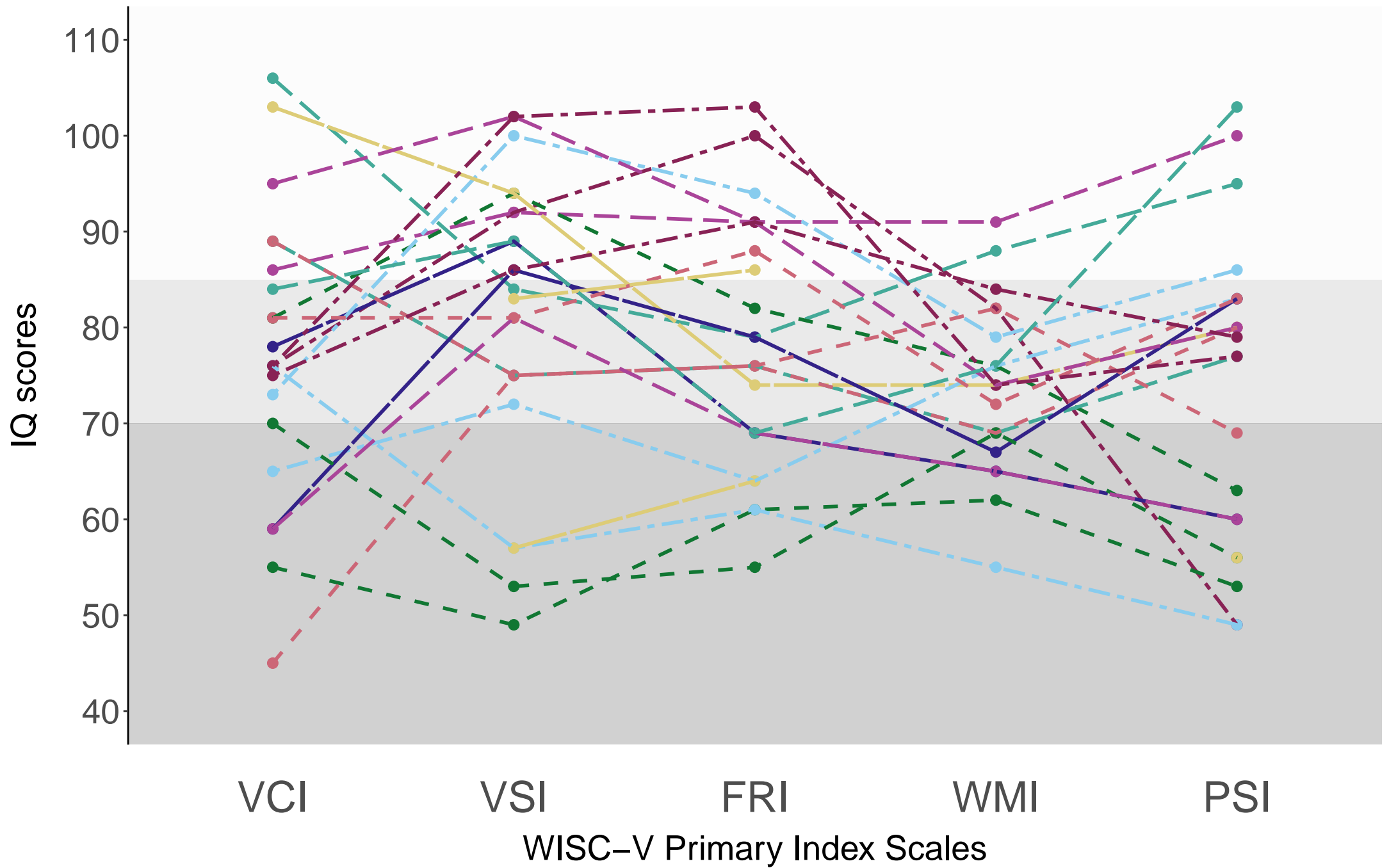

Supplement: Supplementary file 6 — Additional file 6: Supplementary Figure 2. WISC-V Primary Index Scales across patients [file 11689_2025_9615_MOESM6_ESM.zip › Verbesselt_Supplementary_Figure 2.pdf]

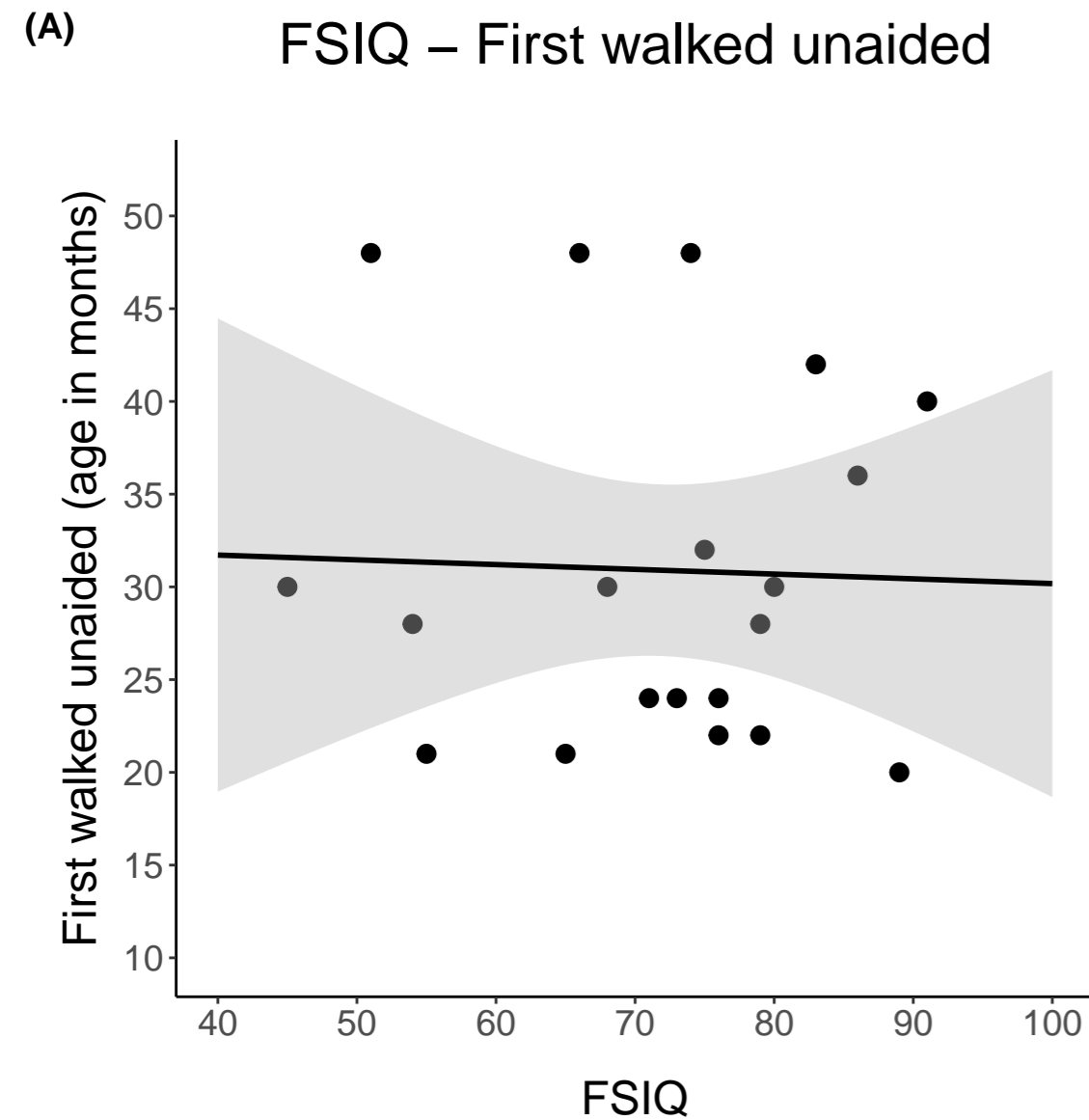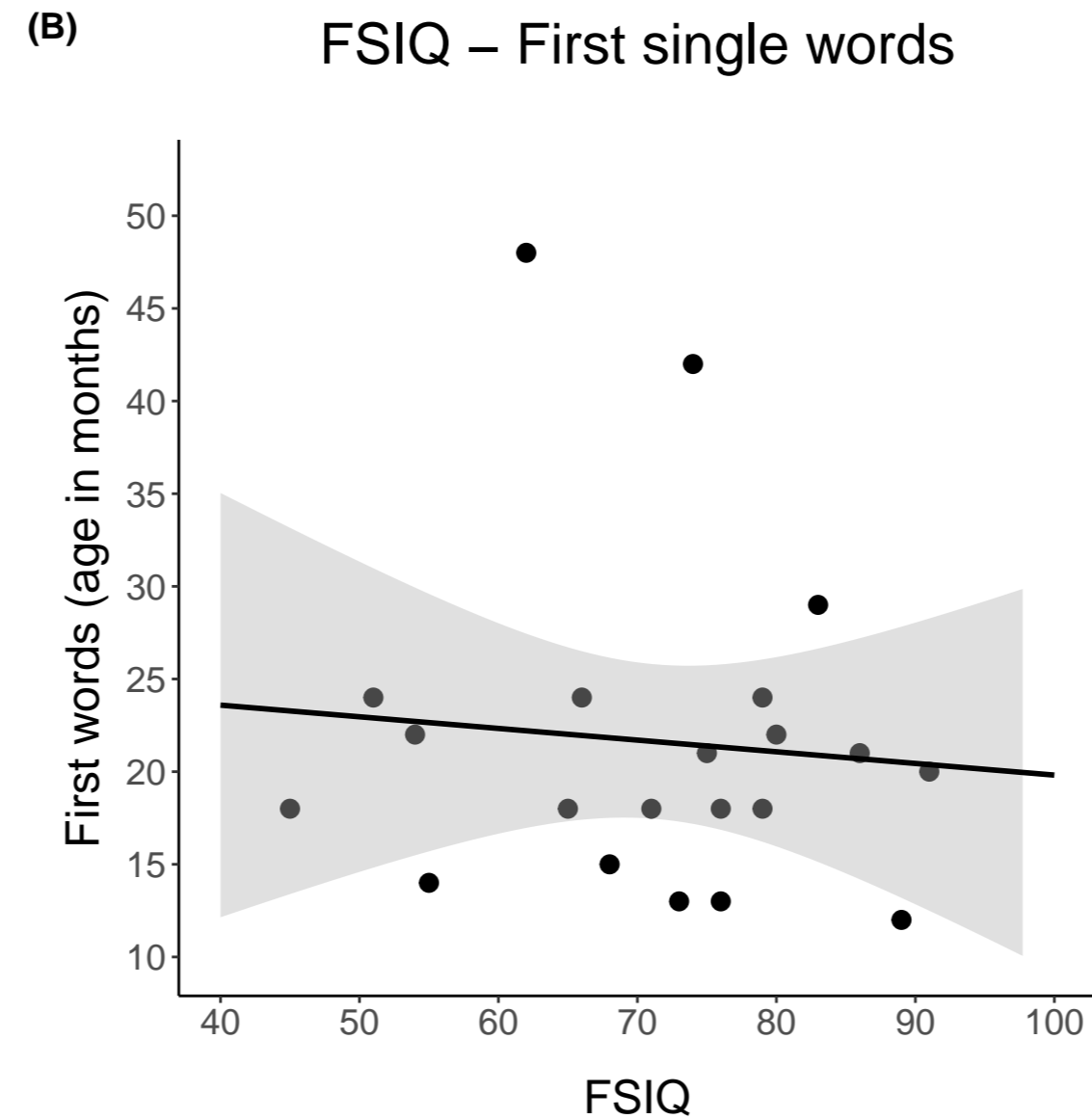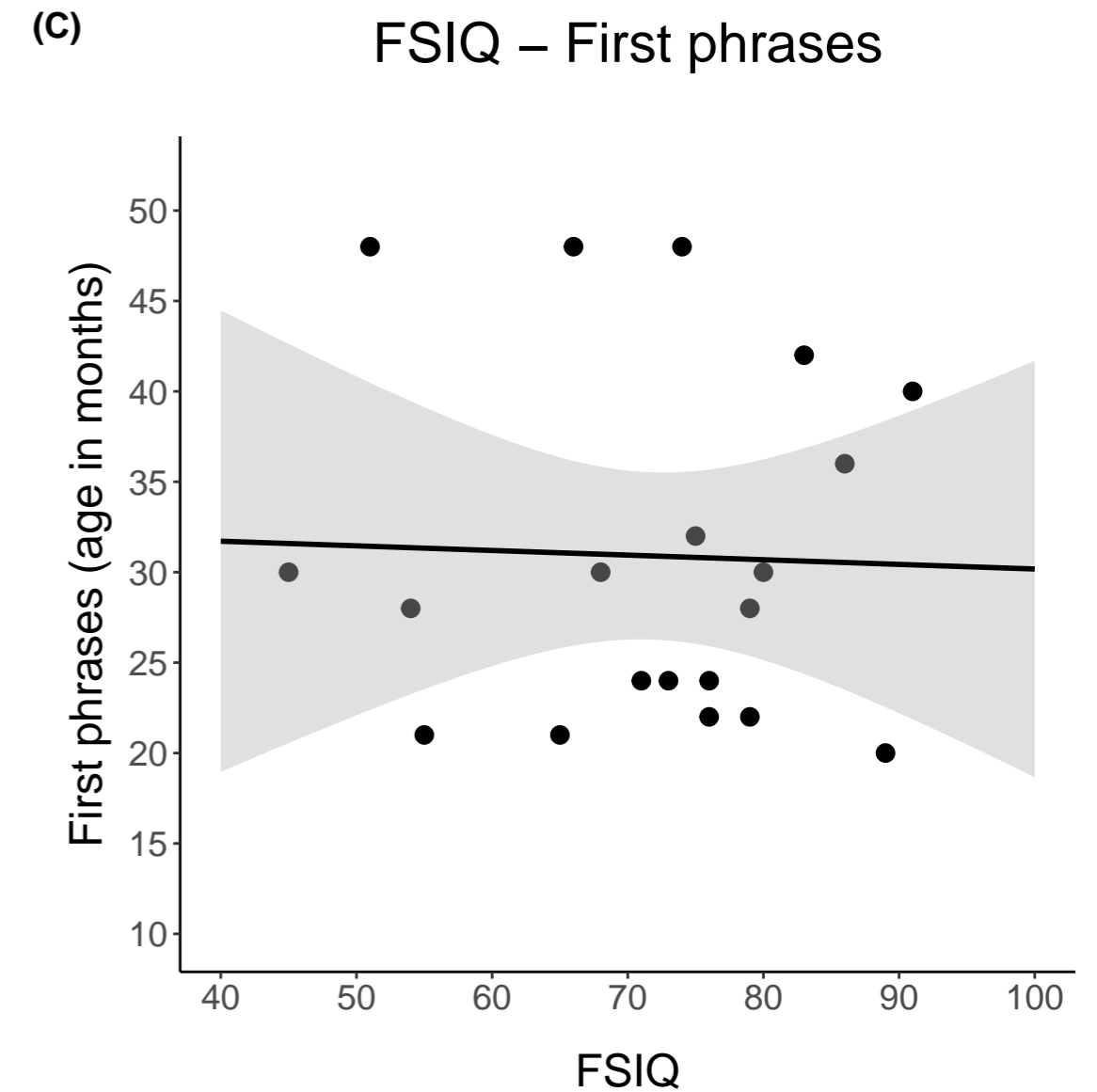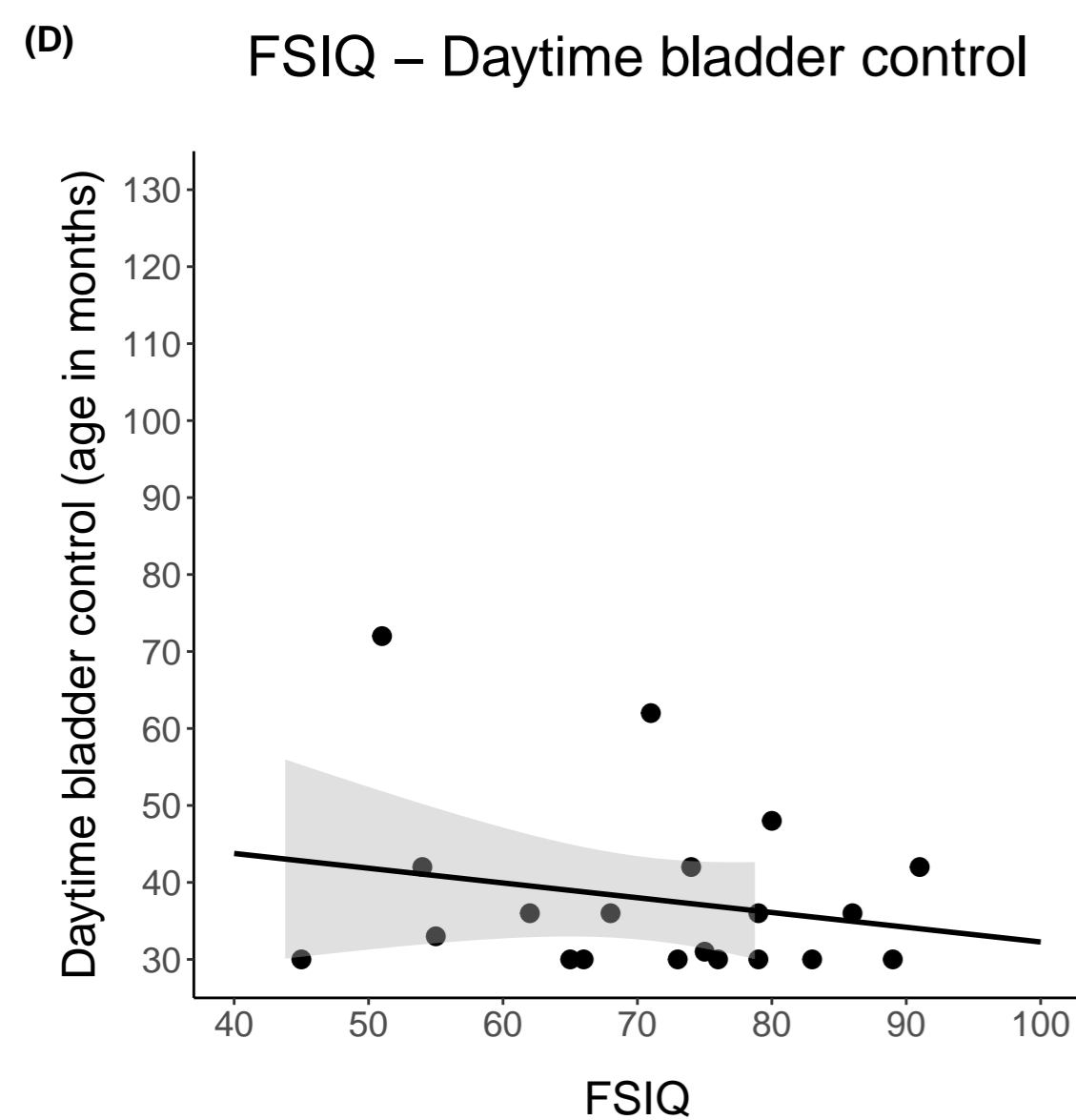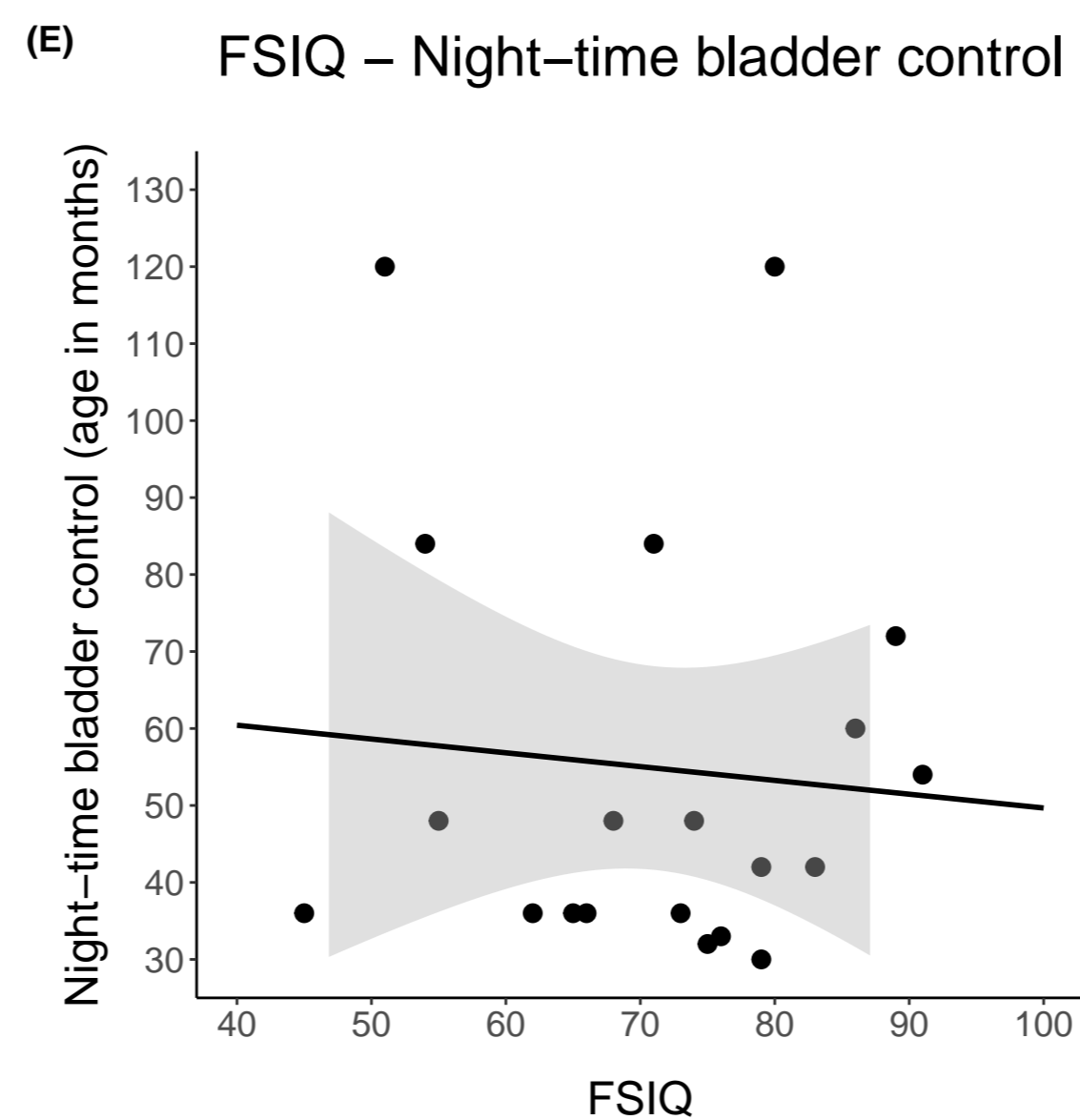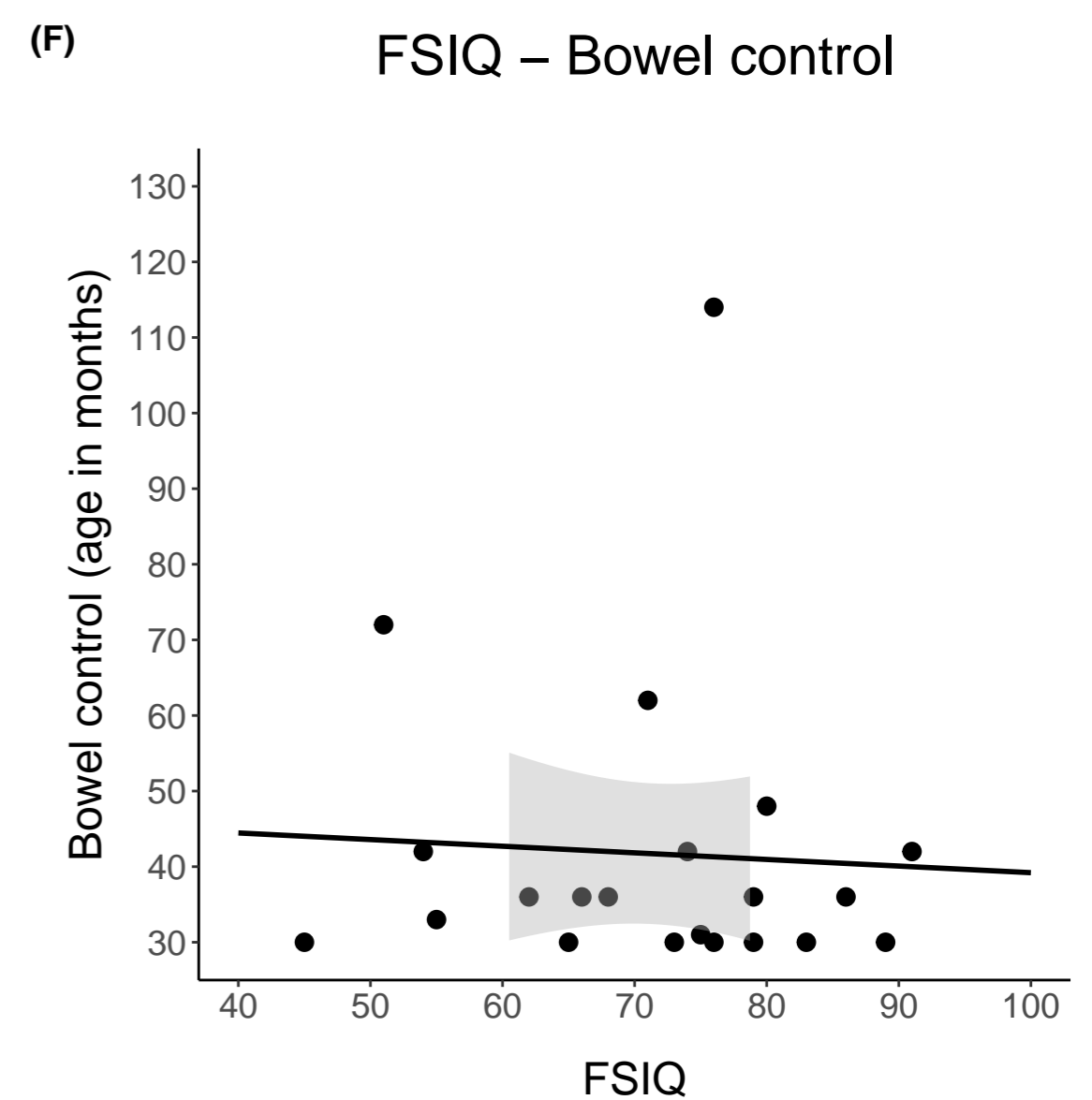

Supplement: Supplementary file 7 — Additional file 7: Supplementary Figure 3. Scatterplots FSIQ and early developmental milestones [file 11689_2025_9615_MOESM7_ESM.zip › Verbesselt_Supplementary_Figure 3.pdf]

(A)

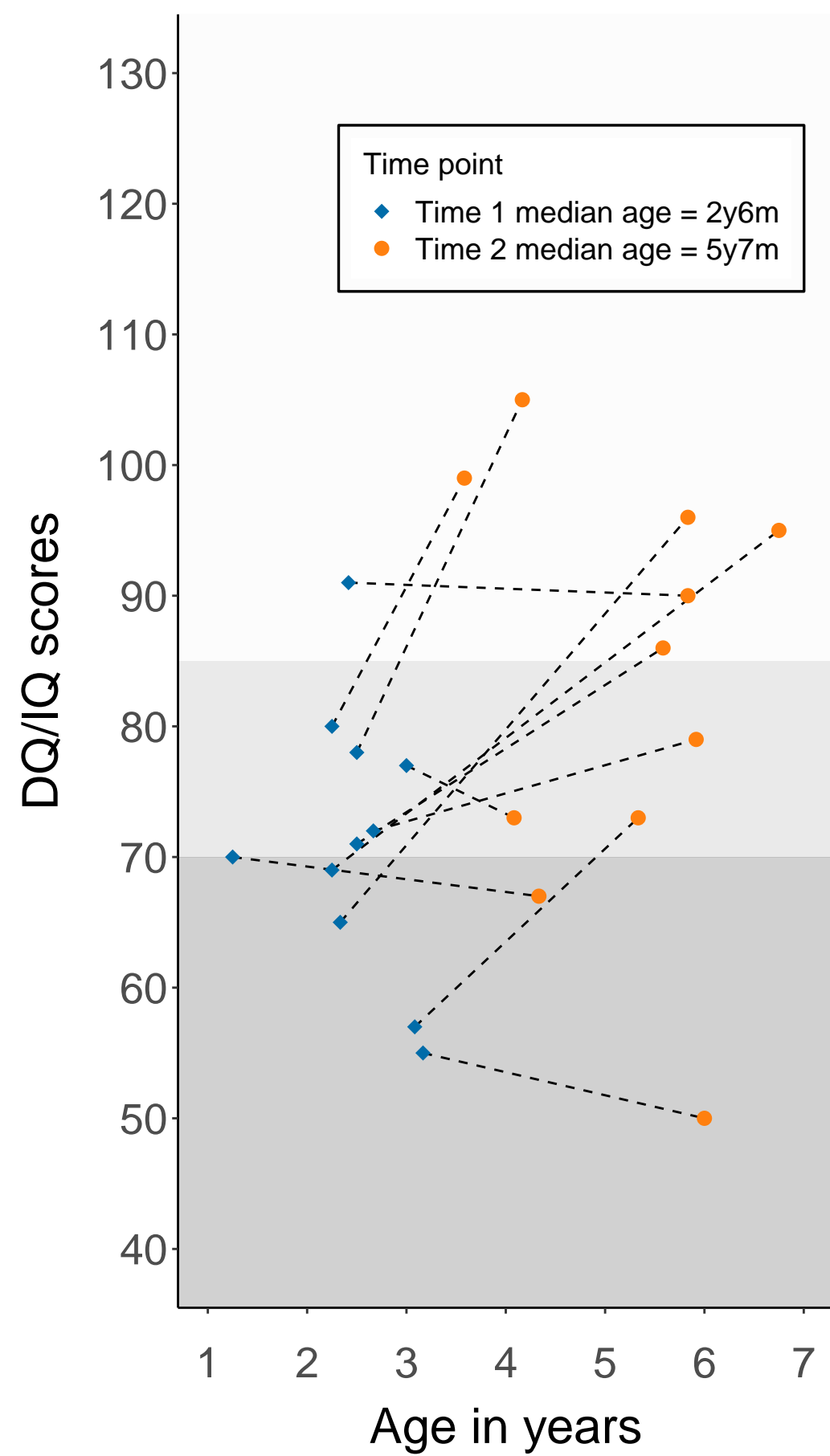

(B)

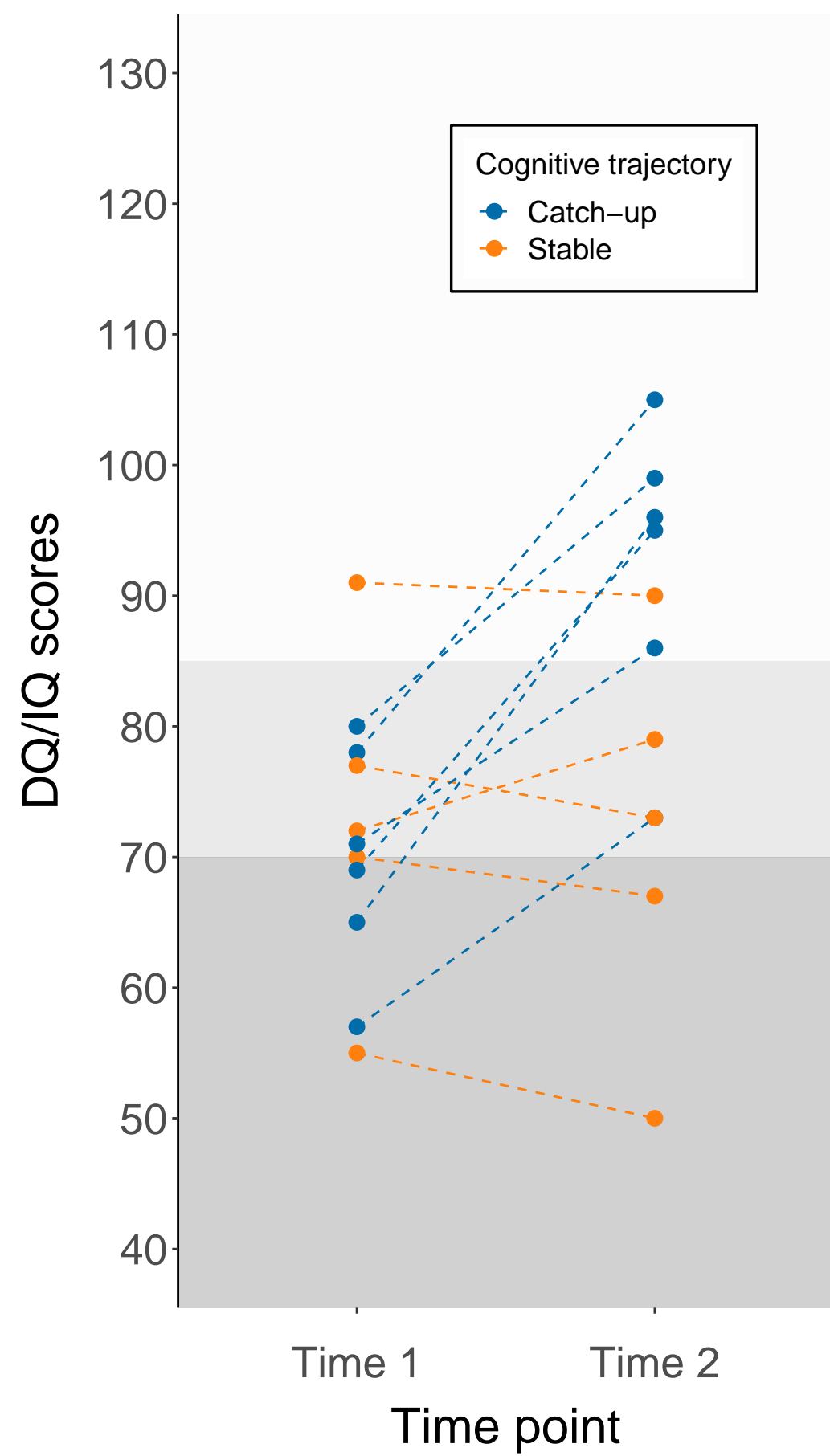

Supplement: Supplementary file 8 — Additional file 8: Supplementary Figure 4. Longitudinal cognitive trajectories in youngest comparison group of children with 16p11.2DS (n=11) [file 11689_2025_9615_MOESM8_ESM.zip › Verbesselt_Supplementary_Figure 4.pdf]
